# Electrospun Nanofiber-Based Biosensors for Foodborne Bacteria Detection

**DOI:** 10.3390/molecules29184415

**Published:** 2024-09-17

**Authors:** Haoming Yang, Song Yan, Tianxi Yang

**Affiliations:** Food, Nutrition and Health, Faculty of Land and Food Systems, The University of British Columbia, Vancouver, BC V6T 1Z4, Canada; hyang24@student.ubc.ca (H.Y.); songyan.nbu@gmail.com (S.Y.)

**Keywords:** contamination, foodborne pathogens, biosensor, electrospinning

## Abstract

Food contamination has emerged as a significant global health concern, posing substantial challenges to the food industry. Bacteria are the primary cause of foodborne diseases. Consequently, it is crucial to develop accurate and efficient sensing platforms to detect foodborne bacteria in food products. Among various detection methods, biosensors have emerged as a promising solution due to their portability, affordability, simplicity, selectivity, sensitivity, and rapidity. Electrospun nanofibers have gained increasing popularity in enhancing biosensor performance. These nanofibers possess a distinctive three-dimensional structure, providing a large surface area and ease of preparation. This review provides an overview of the electrospinning technique, nanofibers and nanofiber-based biosensors. It also explores their mechanisms and applications in the detection of foodborne bacteria such as *Salmonella*, *Listeria monocytogenes* (*L. monocytogenes*), *Escherichia coli* (*E. coli*), *Staphylococcus aureus* (*S. aureus*) and *Pseudomonas putida* (*P. putida*).

## 1. Introduction

Food safety is a critical aspect of public health and well-being. Despite recent advancements in food preservation techniques and food safety measures, notable disease outbreaks associated with foodborne pathogens such as bacteria, fungi, and viruses continue to occur globally. Bacteria, including *E. coli*, *L. monocytogenes*, *Clostridium botulinum* and *Salmonella* spp., are the most common cause of foodborne diseases [1]. Under favourable conditions, these microorganisms can proliferate rapidly in food, resulting in severe health consequences [2]. According to the World Health Organization (WHO), unsafe food is responsible for 600 million cases of foodborne disease and 420,000 fatalities annually, worldwide [3].

The frequent foodborne disease outbreaks present a significant challenge for environmental health management and pose a global health hazard. Reliable detection methods for foodborne bacteria are essential to ensure compliance with established legal and regulatory food safety standards [4].

Table 1 summarizes the conventional and novel methods for detecting foodborne pathogens. There are many conventional methods, such as the culture-based method, biochemical test method, immunological-based method and nucleic acid-based method. For instance, the enzyme-linked immunosorbent assay (ELISA) is an immunological-based method, which offers high selectivity, sensitivity, and assay versatility [5]. However, ELISA shows certain limitations, including a narrow dynamic range, low throughput, modest reproducibility, limited multiplexing capabilities and high cost, and is time-consuming [5]. One of the nucleic acid-based methods, polymerase chain reaction (PCR), is a highly sensitive method for bacteria identification based on their genetic material, without bacterial culture steps. Nevertheless, it is expensive, and it requires sample preparation and specialist equipment (test kit) [6]. Referring to Table 1, despite specificity and selectivity, most conventional detection and identification methods are time-consuming and labour-intensive, and are increasingly considered inadequate for the rapid testing demands of modern food safety [7]. In response to these challenges, there is a growing demand for rapid, accurate, and cost-effective approaches capable of in situ, real-time detection [8].

**Figure 1:**
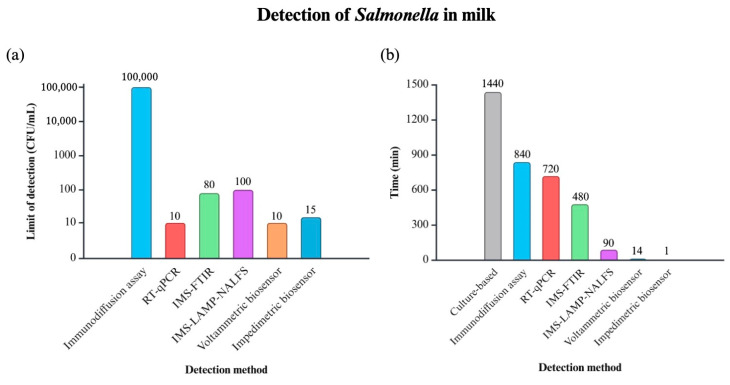
Comparison of different *Salmonella* detection methods in milk, including culture-based method [13], immunodiffusion assay [14], RT-qPCR [15], Immunomagnetic separation–Fourier-Transform Infrared Spectroscopy (IMS-FTIR) [16], immunomagnetic separation–loop-mediated isothermal amplification–nucleic acid lateral flow strip (IMS-LAMP-NALFS) [17], voltammetric biosensor [18], and impedimetric biosensor [19], in terms of (**a**) limit of detection and (**b**) time. It shows that both biosensors demonstrate lower limit of detection and less time compared to other methods.

**Figure 2:**
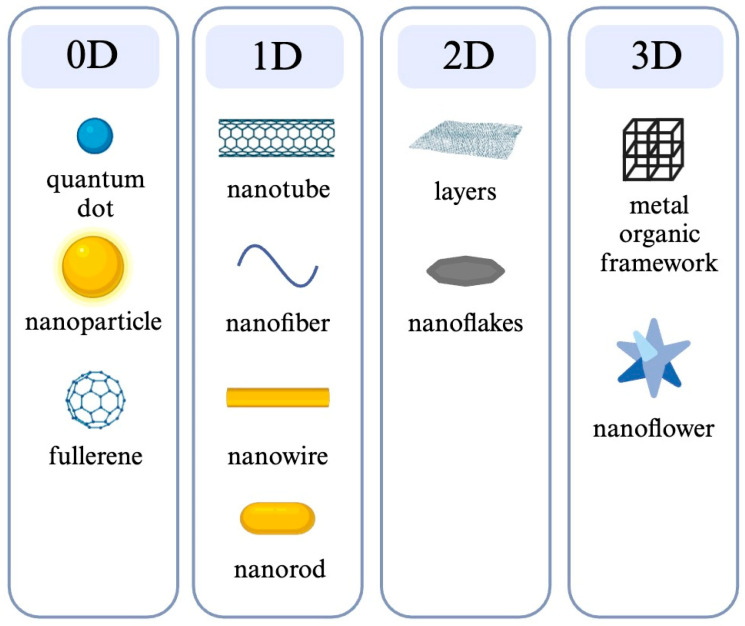
Various morphologies of nanostructured materials range from 0 D to 3 D [12].

Increasingly novel methods have been developed, including the hybridization-based method, the array-based method, spectroscopy, loop-mediated isothermal amplification (LAMP) and the biosensor (Table 1) [9]. Among these, biosensor devices have emerged as a promising solution, gaining substantial popularity in both research and commercial markets. These devices provide numerous opportunities for detecting various analytes in clinical diagnostics, food analysis, and environmental monitoring [10]. Compared to traditional detection methods, biosensors present many advantages, including portability, affordability, simplicity, selectivity, sensitivity, and rapid assay time [11]. For instance, Figure 1 illustrates a comparison of detection time and detection limits between biosensors and other methods—such as culture-based techniques, immunodiffusion assays, RT-qPCR, Immunomagnetic Separation–Fourier-Transform Infrared Spectroscopy (IMS-FTIR), Immunomagnetic Separation–Loop-Mediated Isothermal Amplification–Nucleic Acid Lateral Flow Strip (IMS-LAMP-NALFS), voltammetric biosensors, and impedimetric biosensors—specifically for detecting Salmonella in milk. Both biosensors demonstrate a lower detection time and limit, compared to other methods. Despite these advantages, challenges remain for enhancing sensitivity, specificity, and adaptability in diverse conditions. To address these challenges, researchers have increasingly turned to nanotechnology to advance biosensor capabilities. Nanomaterials, which significantly improve the sensitivity, selectivity, and response time of biosensors, are generally categorized into three dimensions: 0 D (quantum dots, fullerene, and nanoparticles), 1 D (nanofibers, nanotubes, nanowires, and nanorods), 2 D (layers and nanoflakes) and 3 D (nanoflowers and framework) (Figure 2) [12].

Among these nanomaterials, nanofibers have emerged as promising material for biosensors due to their unique properties, such as a high surface-area-to-volume ratio. They have been investigated and employed in creating sensors which are simple to operate, and have enhanced loading capacity, improved sensitivity, and accelerated response times [20]. Various manufacturing techniques have been developed for producing nanofiber membranes, with electrospinning being recognized as the simplest, and relatively cost-effective, method [21]. This paper provides an overview of the principles and applications of electrospun nanofiber-based biosensors, focusing on their use in detecting common foodborne bacteria, including *Salmonella*, *E. coli*, *L. monocytogenes*, *S. aureus* and *P. putida*.

## 2. Biosensors

**Figure 3:**
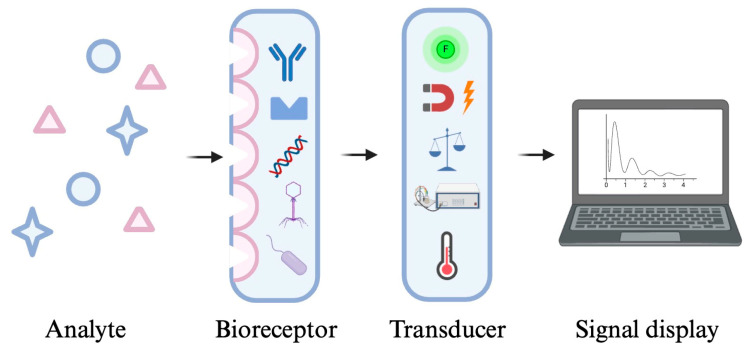
Three basic components of a biosensor: a bioreceptor for reacting with analytes and generating biological signals, a transducer for converting the biological reactions into measurable signals, and a signal display system [20].

Biosensors constitute three components: (1) a bioreceptor, also known as a biological sensing element, (2) a transducer and (3) a signal capture and processing system (Figure 3) [21]. The bioreceptor is used for molecular recognition phenomena. Various biomolecules can serve as bioreceptors, which are typically categorized into five primary groups: enzymes, antibodies, nucleic acids, cells, and bacteriophages. When the bioreceptor reacts selectively with the target analyte, it generates a signal that is transmitted via the transducer to the signal processor. Transduction methods include optical (e.g., absorbance, fluorescence, or luminescence), electrochemical (e.g., voltammetric, potentiometric), electrical, mass sensitive, thermometric, and magnetic approaches [20]. The signal processor then converts the biological reactions into measurable signals and results [22]. Biosensors have been widely developed, with some reaching commercialization and routine application in environmental and agricultural fields, as well as in clinical laboratories and industrial analysis [23]. Among the various types, electrochemical and optical biosensors are particularly prominent in foodborne pathogen detection [24]. Electrochemical biosensors are valued for their high sensitivity, selectivity and fast response [25]. A typical electrochemical biosensor consists of several key components: an analyte, a bioreceptor, an electrochemical transducer, electronics, and a result display [25]. The functioning of the electrochemical biosensor relies on the interaction between the bioreceptor and the analyte on the transducer surface, resulting in observable electrochemical signals, such as voltage, current, impedance, and capacitance, enabling both quantitative and qualitative analysis of the analyte [26]. Electrochemical biosensors can be further categorized, based on their transduction principle, into types like amperometric, potentiometric, voltammetric, conductometric, and impedimetric biosensors [27]. On the other hand, an optical biosensor detects analytes through optical phenomena triggered by the interaction between the receptor and the analyte. These sensors can utilize various optical effects, including absorbance, luminescence, fluorescence (either emission or quenching), or surface plasmon resonance [20]. For instance, fluorescence biosensors detect analytes by recording changes in fluorescence intensity, offering high sensitivity, specificity, resistance to light scattering, and ease of use, making them ideal for detecting biomolecules or metal ions [28]. Similarly, colorimetric biosensors, a subset of optical sensors, detect specific analytes through colour change observation [29]. Optical biosensors are valued for their affordability, compact size, and real-time detection capabilities. However, they may have limitations such as lower sensitivity, the requirement for specialized equipment, and, occasionally, longer detection times [30].

## 3. Electrospinning and Nanofibers

### 3.1. Nanofiber

Nanofibers are typically defined as ultra-fine solid fibers with diameters of 1000 nm or less, specifically those under 100 nm [31]. These nanofibers can be fabricated from a variety of polymers, including keratin, collagen, silk fibroin, cellulose, gelatin, (poly)lactic acid (PLA), (poly)lactic-co-glycolic acid (PLGA), (poly)ethylene-co-vinylacetate) (PEVA), and polysaccharides (alginate and chitosan) [32]. Nanofibers can be categorized by source material (organic, inorganic, carbon, and composite) and structure (nonporous, mesoporous, hollow, and core-shell) [33]. Besides their nanoscale sizes, nanofibers have garnered significant interest due to their large surface area, porosity, ease of fabrication, flexibility, and favourable chemical, physical, and mechanical properties. Additionally, nanofibers offer the potential for morphological control and can be easily functionalized to gain additional properties required for various applications [34]. Thus, nanofibers have found extensive use across diverse fields, including energy generation, production, storage, environmental protection and improvement, tissue engineering, pharmaceuticals, and biomedical fields [33].

### 3.2. Electrospinning

There are various ways to generate nanofibers, including drawing, template synthesis, phase separation, self-assembly and electrospinning [35]. Among these, electrospinning stands out as the preferred choice for large-scale industrial production of nanofibers, particularly through solution and melt electrospinning techniques [35,36]. This preference is attributed to the technique’s ease of handling, processing convenience, cost-effectiveness, simplicity, and reproducibility in fiber production. Electrospinning also minimizes solution consumption and controls precisely fiber diameter [37]. Moreover, electrospinning allows for customization of the diverse structure and morphology of the resulting nanofibers [38]. For instance, various structures (solid, hollow, core-shell, porous, and janus), as well as different orientations (random, aligned, and layer-by-layer deposition), can be designed and produced through the electrospinning technique or in conjunction with certain post-modification methods [39,40,41].

**Figure 4:**
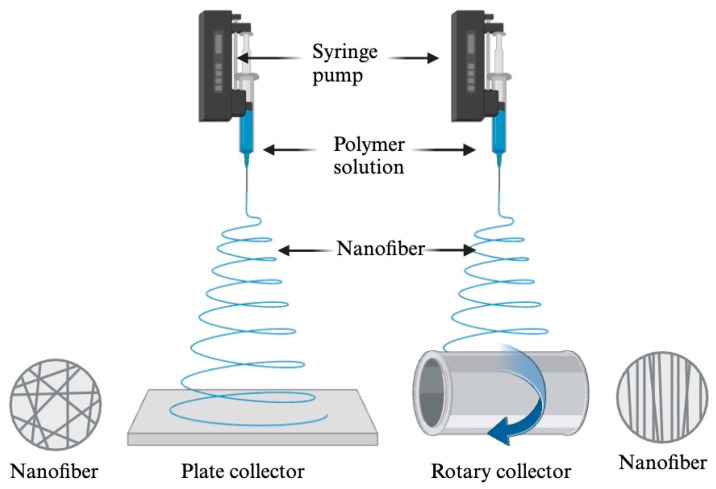
Electrospinning setup with a plate collector and a rotary collector [20].

A typical electrospinning setup consists of several key components: (1) a high-voltage power supply (direct current or alternative current), (2) a syringe containing a solution of the desired polymer, (3) a spinneret (often a needle or a pipette tip), and (4) a collecting platform (plate or rotary). Figure 4 illustrates schematically the single-needle electrospinning process, which is simple and easy to reproduce. Firstly, fill the syringe with the prepared polymer solution and load it into the syringe pump. Then, attach the needle to the syringe and connect it to the high-voltage power supply. The grounded collector should be placed at a fixed distance (usually 10–20 cm) from the needle [42]. The basic principle of this technique is electrostatic interaction [37]. When a high voltage (~10–30 kV) is applied between the spinneret and collector, electrostatic forces cause the charged droplet at the spinneret’s tip to deform into a conical-shaped droplet known as a Taylor cone. As the voltage reaches a critical level, a spinning jet is produced from the tip of the Taylor cone. Initially, the jet extends linearly, but it soon undergoes vigorous whipping motions, due to unstable bending. The high charge density on the collector, placed at an optimized distance, attracts the jet, causing it to stretch into finer diameters, elongate, and solidify quickly, due to solvent evaporation. This process results in the deposition of uniform solid nanofibers on the collector [42].

Electrospinning is influenced by various significant factors, including applied voltage, solution concentration, viscosity, conductivity, solvent types, and the distance between the electrospinning needle and the collector, as well as ambient conditions such as relative humidity and temperature [43]. These parameters play a crucial role in determining the outcomes of the electrospun nanofibers. Therefore, optimizing these electrospinning parameters is essential for achieving the desired outcomes [43].

### 3.3. Electrospun Nanofibers in Biosensors

**Figure 5:**
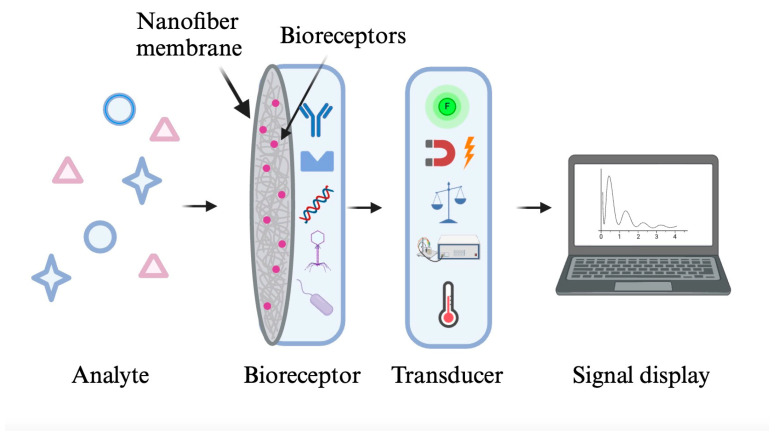
A schematic diagram of the nanofiber membrane-based biosensor [21].

In recent years, the combination of nanomaterials with various biosensors has led to the continuous development of nano-biosensors [44]. Electrospinning techniques (Figure 4) are used to create nanofiber membranes, onto which biological materials (bioreceptors) are applied and immobilized (Figure 5) [21]. During the operation, these biological substances interact with the target analytes, which are then converted into signals by the signal transmitter [45].

**Figure 6:**
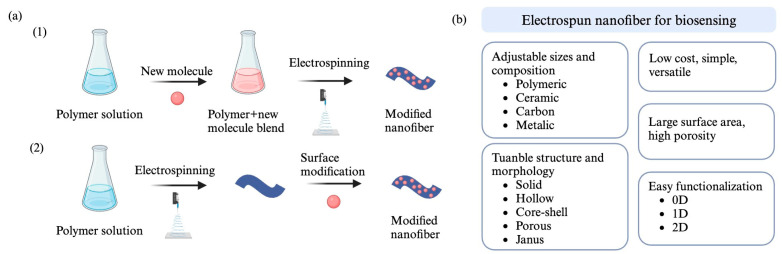
(**a**) Functionalizing nanofibers through two approaches: (1) direct incorporation; and (2) surface modification [46]. In direct incorporation, new molecules and polymer solution are mixed prior to electrospinning. In surface modification, new compounds are deposited onto electrospun nanofibers. (**b**) Advantageous features of electrospun nanofibers for biosensing applications [10].

Figure 6a illustrates two approaches to nanofiber functionalization: direct incorporation and surface modification [46]. In direct incorporation, new molecules are added to the polymer solution before electrospinning, allowing them to be uniformly embedded within the structure of the resulting nanofibers [47]. For instance, Zhang and his colleagues incorporated active metal nanoparticles into an electrospinning solution, to improve electrochemical performance [48]. This method is simple and broadly applicable, but it has limitations, such as reduced utilization of the sensing unit and susceptibility to easy separation [49]. Conversely, in surface modification, electrospun nanofibers are used as templates onto which new compounds are deposited [47]. This technique involves coating the nanofibers with functional molecules or polymers, using methods such as layer-by-layer assembly or spray-coating [50]. For instance, layer-by-layer treatment allows the physical immobilization of oppositely charged macro-molecules to build up the coating [50]. This approach facilitates strong bonding between the sensing unit and the nanofiber, but also has the drawback of limited sensing-unit utilization [49].

Figure 6b highlights the numerous advantages of nanofiber-based biosensors compared to conventional biosensors. These advantages include a high specific-surface area, high porosity, adjustable voids, high responsiveness, small fiber diameter, enhanced sensitivity, a broad range of detectable targets, and cost-effectiveness [10]. The large surface area of nanofibers allows for high loadings of biomolecules and efficient interaction with analytes, thereby enhancing biosensor sensitivity [21]. Additionally, nanofibrous membranes are commonly used as functional support matrices for immobilizing bioreceptors, such as proteins, enzymes, antibodies, aptamers, whole cells, and synthetic molecularly imprinted polymers (MIPs) [10].

## 4. Application of Electrospun Nanofibers for Detecting Foodborne Bacteria

Electrospun nanofibers have emerged as a promising tool for the detection of foodborne bacteria, due to their unique structural and functional properties. Consequently, electrospun nanofibers are being increasingly utilized in food safety applications to detect pathogens like *Salmonella*, *E. coli*, *L. monocytogenes*, *S. aureus* and *P. putida*, ensuring the safety and quality of food products. Table 2 summarises different types of electrospun nanofiber-based biosensors for the detection of foodborne bacteria.

### 4.1. Salmonella

*Salmonella* is a rod-shaped Gram-negative bacteria commonly related to food outbreaks. Traditional methods for detecting Salmonella are often time-consuming and labour-intensive. To address these limitations, recent research has increasingly focused on nanofiber-based biosensors, with electrospun nanofiber sensors emerging as particularly effective, due to their enhanced sensitivity and faster detection times. Thiha et al. integrated electrospinning and photolithography techniques to develop carbon nanowire sensors with sizes below 100 nm [51]. Carbon materials are known for their rapid electron transfer kinetics, exceptional conductivity, and ease of biofunctionalization. The sensor was integrated with a microfluidic chip for label-free chemical resistive biosensing. They used the carboxylic groups on carbon nanowire surfaces to immobilize amine-terminated aptamers onto suspended nanowires, utilizing carbodiimide crosslinker chemistry. The results demonstrated the sensor’s high speed, specificity, sensitivity and low sample-volume requirement (5 µL). It enabled Salmonella detection with a limit of detection (LOD) of 10 CFU/mL and a response time of only 5 min [51]. This assay time (5 min) is much shorter than PCR (3 h), loop-mediated isothermal amplification (LAMP) (3 h), Carbon nanotube field-effect transistor (CNT-FET) electronic sensor (1 h) and Lab-on-Disk LAMP florescent microdevice (1 h) [67,68,69,70]. The LOD (10 CFU/mL) is also the lowest, compared to PCR (10^3^ CFU/mL), LAMP (10^2^ CFU/mL), the CNT-FET electronic sensor (10^2^ CFU/mL) and the Lab-on-Disk LAMP florescent microdevice (2.7 × 10^4^ CFU/mL) [67,68,69,70]. However, due to the stochastic nature of the electrospinning process, further enhancements are needed to precisely control the positioning and morphology of the nanowires [51].

**Figure 7:**
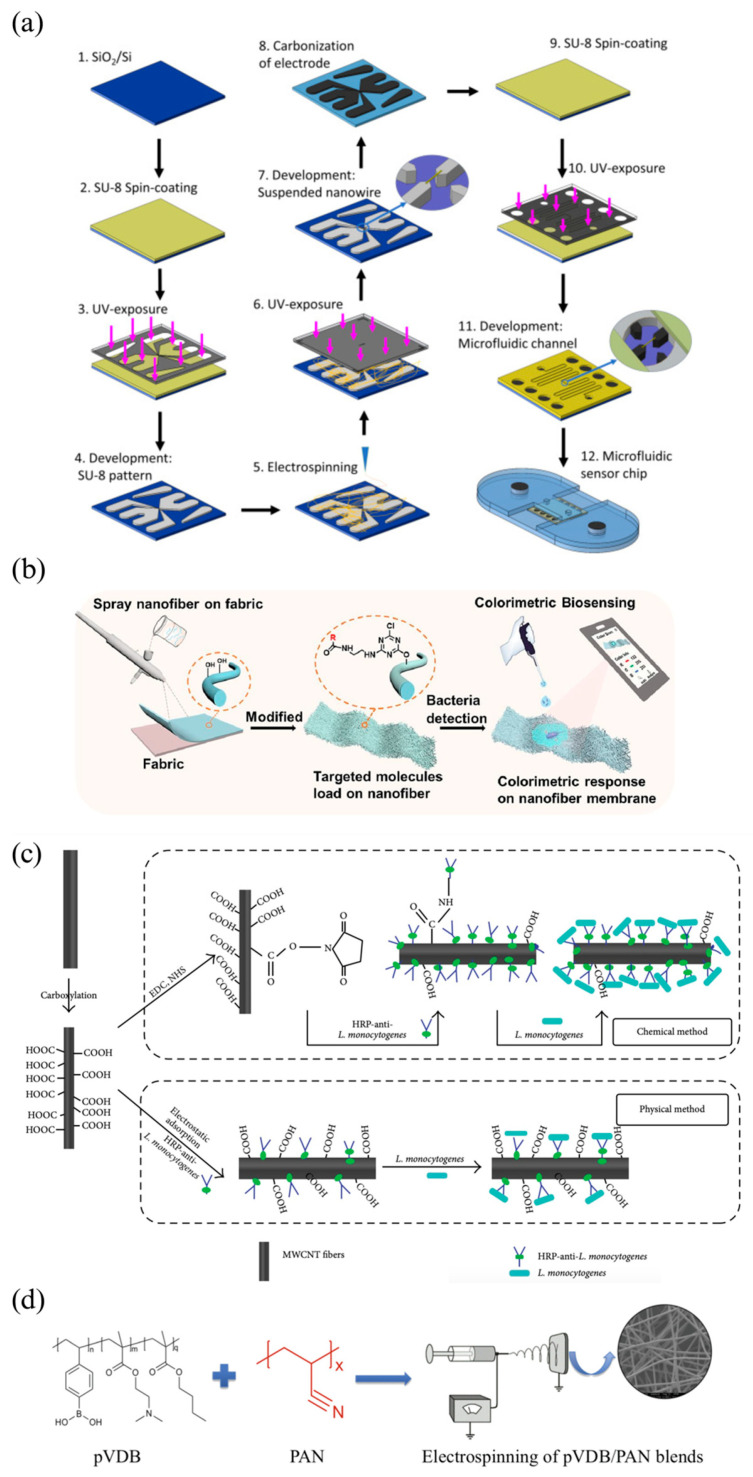
(**a**) Fabrication steps of carbon nanowire biosensor for *Salmonella* detection [52]. (**b**) A nanofiber-based colorimetric platform for *E. coli* detection [56]. (**c**) Immunoelectrode fabrication by physical and chemical immobilization methods for *L. monocytogenes* detection [62]. (**d**) Electrospun nanofiber membranes prepared from PAN and pVDB for *S. aureus* and *P. putida* detection [64].

Another study also uses carbon nanofibers, and the researchers developed an aptasensor based on Chi-electrospun carbon nanofibers/Au NP-decorated pencil graphite electrode (GE) for electrochemical detection of Salmonella in milk (Figure 7a) [52]. The carbon nanofibers were used to modify the electrode surface, facilitating the immobilization and adsorption of biomolecules, while simultaneously enhancing conductivity, sensitivity, and detection limits for Salmonella [52]. PCR is unable to detect *Salmonella* with concentrations lower than (10^2^ CFU/mL). Compared to the PCR technique, the developed aptasensor demonstrated exceptional selectivity and sensitivity, even in real samples, with a LOD of 1.223 CFU/mL [52]. To assess selectivity, the engineered GE was incubated with 10³ CFU/mL of various controls, including *E. coli*, *P. aeruginosa*, and *S. aureus*. The differential pulse-voltammetry results showed a much lower signal current, compared to *Salmonella*, indicating high specificity for *Salmonella* detection [52].

In addition to carbon nanofibers, Guler Gokce et al. fabricated an impedimetric DNA biosensor using electrospun PU/P3ANA nanofibers [53]. P3ANA exhibits excellent electrochemical activity across a wide pH range, along with favourable mechanical properties and processability. Electrospinning allows the fabrication of nanofibers for ideal properties, through utilizing various electrospinnable polymer materials. In this study, PU was chosen for its superior fiber-forming abilities and mechanical strength, enhancing the stability and durability of the biosensor when combined with P3ANA [53]. The DNA probe was covalently attached to PU/P3ANA nanofibers via carboxyl groups on the nanofibers. Then, the PU/P3ANA nanofibers served as a transducer, due to their high surface-area-to-volume ratio. The biosensor exhibited high selectivity (8.17 kΩ/μM) and sensitivity to single-base mismatch mutations, and demonstrated stability over one month [53].

### 4.2. E. coli

*E. coli* is a Gram-negative, facultative anaerobic bacterium normally found in the intestines of humans and animals, but certain strains can cause severe foodborne illnesses. Recent studies have demonstrated the effectiveness of nanofiber-based biosensors in detecting *E. coli* at low concentrations, providing a powerful tool for preventing contamination and ensuring public health. Li et al. prepared a colour indicator film using electrospun PLLA and anthocyanin nanofiber, for performing bacterial detection at low levels in meat and seafood. The film was able to sense both Gram-positive and Gram-negative bacteria (*E. coli* and *L. monocytogenes*) at concentrations as low as 10^2^ CFU/mL [55]. Similarly, Zhang et al. presented a colorimetric platform with nanofiber membranes loaded with target molecules (fluorescent and chromogenic substrate) through chemical modifications (Figure 7b) [56]. During the metabolic process, *E. coli* secretes *β*-glucuronidase, which triggers the functionalized nanofiber membrane to produce biological signals, resulting in a colour change from colourless to fluorescent blue or green. The colour was observed for the quantitative and qualitative detection of *E. coli* concentration. The highly specific nanofiber exhibited no false positives with the presence of ions or pH interference [56]. Notably, this device can achieve on-site *E. coli* detection by integrating with a smartphone app, which demonstrates great potential for food safety testing [56]. Another colorimetric biosensor based on electrospun PDA nanofiber by Bhattacharjee et al. achieved selective detection of Gram-negative bacteria (such as *E. coli*) via a quick colorimetric change from blue to red [57]. The selectivity primarily differs in the structural characteristics of Gram-positive and Gram-negative bacteria, such as disparities in cell outer-layer thickness, along with the diverse extracellular polymeric substances (EPSs) released by distinct bacterial strains [57].

In addition to optic sensors, electrochemical biosensors are also commonly used for *E. coli* detection. Shaibani et al. developed a portable, light-addressable potentiometric sensor (LAPS) for *E. coli* detection in water and orange juice, respectively [55]. The LAPS is integrated with a sensing layer, using electrospun PAA/PVA hydrogel nanofibers. Changes in the pH of the media are detected by the LAPS system, based on the sugar molecule fermentation of *E. coli* and the production of acidic products such as lactates and acetates, which increase the acidity of the surroundings. The swelling and shrinking of nanofibers can indicate pH changes in the medium, affecting the LAPS photocurrent signal. Additionally, the selectivity toward *E. coli* was achieved, as *E. coli* has a higher affinity toward mannose, compared to *Salmonella Typhimurium* (*S. Typhi*), so the pH is lower for *E. coli* than for S. Typhi in the same period [59]. The results showed the nanofiber-integrated LAPS could detect *E. coli* of 10^2^ CFU/mL in orange juice in less than 1 h, and *E. coli* of 20 CFU/mL in water [59]. This LOD is similar to using impedance spectroscopy in water samples at pH 7 to 9 (10^2^ CFU/mL) and a microfluidic chip in juice samples (10^2^ CFU/mL) [71,72]. However, the nanofiber-integrated LAPS does not require orange juice dilution before testing, and it is not affected by the complex nature of orange juice [59].

Luo et al. utilized an electrospun nitrocellulose nanofibrous membrane to make a direct-charge-transfer biosensor [60]. This membrane directs pathogens to the immobilized secondary antibody, facilitating the formation of a sandwich complex. This complex accumulates and creates an electron transport path across the silver electrodes. The electrospun nitrocellulose membrane, with its distinctive porous nanostructure and large surface area, increases the mass transfer rate and offers more capillary channels. This enhancement resulted in improved immunoreaction rates and better separation effects. Following capillary flow equilibrium, the direct charge transfer between the electrodes correlates with the captured sandwich complex, enabling the determination of pathogen concentration. The biosensor exhibited a linear detection response for *E. coli* and bovine viral diarrhea virus (BVDV), with an LOD of 61 CFU/mL and 10^3^ CCID/mL in a rapid 8 min detection process [60]. Detecting multiple pathogens at the same time is particularly valuable in complex environments where the presence of various microorganisms might pose risks, such as in food safety, clinical diagnostics, and environmental monitoring. In addition, the functionalization process can be adapted to target other microbial or viral organisms, by appropriately modifying the antibodies used. This is also significant, as researchers can tailor the biosensor to detect a wide range of microbial or viral organisms.

### 4.3. L. monocytogenes

*L. monocytogenes* is a serious foodborne pathogen known for causing listeriosis, a potentially fatal infection particularly dangerous to pregnant women, newborns, the elderly, and immunocompromised individuals. Due to its resilience and ability to thrive in a variety of environments, rapid and accurate detection of *L. monocytogenes* is essential for food safety. Lu and his colleagues fabricated an enzyme-labelled amperometric immunosensor based on MWCNT fibers (Figure 7c) [62]. These MWCNT fibers consist of highly aligned and multi-walled carbon nanotubes with interlamellar distances, contributing to high tensile strengths and electrical conductivities. The detection of *L. monocytogenes* was achieved by monitoring the change in direct electrochemical signal resulting from the binding of antigen and horseradish peroxidase-labelled antibody. The immunosensor enabled the detection of *L. monocytogenes* as low as 1.07 × 10^2^ CFU/mL in the milk sample and 1.51 × 10^3^ CFU/mL in the milk sample with mixed bacteria. The use of nanofibers optimized the electrical conductivity, biocompatibility, and electron transfer rate of immunosensors. The developed immunosensor demonstrated high specificity, storage stability and reproducibility [62].

Another study used a TiO_2_ nanowire bundle microelectrode-based impedance immunosensor to detect *L. monocytogenes* [63]. Similar to the previous study, monoclonal antibodies (positively charged) were attached to the surface of the TiO_2_ nanowire bundle (negatively charged) to target *L. monocytogenes*, specifically. The change in impedance caused by the formation of the nanowire–antibody–bacteria complex was measured, and used to determine the bacterial count. The immunosensor achieved the detection of *L. monocytogenes* at a concentration of 4.7 × 10^2^ CFU/mL, with a total detection time of 50 min [63]. The TiO_2_ nanowire bundle microelectrode-based impedance immunosensor achieved lower LOD for L. monocytogene detection, compared to other conventional immunoassay methods or immunosensor methods, such as direct ELISA (10^6^ to 10^8^ CFU/mL), SPR biosensor (2 × 10^2^ CFU/mL) and fiber-optic immunosensor (4.3 × 10^3^ CFU/mL) [73,74,75].

### 4.4. S. aureus and P. putida

*S. aureus* is a Gram-positive, spherically shaped bacterium. It is a major pathogen responsible for a variety of infections, ranging from minor skin infections to life-threatening diseases such as pneumonia and sepsis. *P. putida* is Gram-negative and rod-shaped. Though less harmful to humans, it is recognized as a spoiler of fresh foods under cool conditions. The use of electrospun nanofibers in biosensors provides an innovative approach to achieving high sensitivity and specificity in detecting these bacteria. For instance, Jennifer et al. (2017) developed electrospun nanofiber membranes using a blend of polyacrylonitrile (PAN) and pVDB, which were utilized as fluorescent bacterial biosensors. The detection mechanism was based on the reversible formation of boronate esters with the diol-rich saccharide components present on bacterial membranes (Figure 7d) [64]. The interaction allows the system to effectively bind with bacteria, facilitated by the presence of a fluorescent reporter tag. Maximum fluorescence intensity was observed for *S. aureus* and *E.coli* after 24 h of contact. However, the membranes ceased to function after 8 h of exposure to *P. putida*, as the formation of bacterial biofilms blocked the membrane surface, thereby disrupting fluorescence signal reading [64]. This suggests that electrospun nanofibrous membrane has the potential for simple bacterial detection during the early stage of microbial colonization. In another study, Kim et al. (2020) developed electrospun PDA/PU nanofibers, which effectively detected Gram-positive and Gram-negative bacteria through a visible colour change. The detection was made possible due to the bacterial-blocking properties of the nanofibers [66]. When exposed to bacteria, the phospholipids in *S. aureus* interact with PDA, resulting in the shortening of the PDA main chain and a transition in the binding mode from in-plane to twisted [66]. This interaction triggered a colour change from blue to red, which was observed in a number of *S. aureus* bacteria 45 × 10^2^ CFU/mL. The degree of color change allowed for quantitative analysis of bacterial concentration. Furthermore, beyond food bacteria detection, the PDA/PU nanofiber web demonstrates the potential to be applied to a mask or filter, due to its blocking properties.

## 5. Future Outlook

The potential for commercializing electrospun nanofiber-based biosensors in food safety is promising, yet it presents challenges that need to be addressed to transition from research to market. While many studies highlight the low cost of producing electrospun nanofibers, there is often a lack of detailed pricing information and direct comparisons with existing detection methods. In addition, not every research study specifies the amount of time used for bacteria detection. Rapid detection is essential, as it can prevent contaminated products from reaching consumers and reduce the risk of widespread foodborne-illness outbreaks. As the trend toward portable, user-friendly biosensors continues, integrating electrospun nanofiber-based biosensors into compact, handheld devices is a promising solution. These devices could enable on-site bacterial detection in food production facilities, restaurants, and households, providing real-time results and reducing the reliance on laboratory testing. Some researchers have developed biosensors that can detect multiple pathogens at the same time. For instance, the direct-charge-transfer biosensor developed by Luo et al. can detect both *E. coli* and BVDV [60]. This advancement represents a significant leap forward in biosensor technology, allowing for comprehensive and efficient pathogen detection in a single test. The ability to target multiple microorganisms at once is particularly beneficial in environments where a variety of pathogens could be present, such as in food safety testing, clinical diagnostics, and environmental monitoring.

In addition to efficiency and cost, there is a need for environmental consideration. Some existing electrospun nanofibers utilize non-degradable materials such as PAA, which can lead to the environmental concern of microplastic pollution [36]. As a result, sustainable advancements in biodegradable polymeric materials have been developed, such as chitosan, cellulose, proteins, and amino acid polymers, as well as reusable electrospun nanofibers [36]. These materials naturally break down over time, reducing environmental impact compared to non-degradable synthetic polymers.

Electrospinning also has disadvantages, such as difficulty in achieving uniformity and controlled alignment, making it unable to be used for large-scale manufacturing [76]. Some nanofibers may exhibit insufficient electrical conductivity, thereby restricting their suitability for applications requiring high electrical sensitivity [36]. Additionally, nanofibers are prone to fragility and breakage, which can potentially compromise the durability and robustness of the sensors [36]. However, it is noticeable that the advantages and disadvantages can vary, depending on the specific application and different types of nanoparticles [76]. Moreover, while most studies are performed in water or lipid phases, there is a critical need for real food sample detection. Foods present complex matrices that vary widely in solubility (water-soluble, oil-soluble), acidity, and alkalinity, posing significant challenges for accurate and reliable detection [77]. Future research should focus not only on improving the technical aspects of these biosensors, but also on providing cost analyses and developing strategies for scaling up production and integrating these sensors into the food safety industry.

## 6. Conclusions

This paper provides a comprehensive overview of electrospun nanofiber-based biosensors for bacteria detection, including *Salmonella*, *E. coli*, *L. monocytogenes*, *S. aureus*, and *P. putida*. Electrospun nanofibers offer numerous benefits, such as large surface area, porosity, simple fabrication, flexibility, and stability. These properties enhance sensor performance, resulting in improvements in LOD, selectivity, sensitivity, repeatability, responsiveness, and short recovery time. Despite these benefits, there are still challenges for electrospun nanofibers, such as lack of durability, uniformity for industrial application, and not being biodegradable. Detecting bacteria in real food samples is also difficult, as food mediums can differ significantly. Future research and development efforts should focus on addressing these challenges to achieve more accurate, cost-effective, rapid, and environmentally friendly on-site testing solutions.

## Figures and Tables

**Table 1:** Traditional and novel detection methods of foodborne pathogens [9].

	Detection Method	Description	Types	Advantages	Disadvantages
Conventional methods	Culture-based method	Traditional method where bacteria are grown on selective media to detect and quantify viable organisms. Colonies are counted after incubation to determine bacterial load.	Pre-enrichment, selective enrichment	Cost-effective, selective and distinctive	Time-consuming (18–24 h or several days), labor intensive
	Biochemical test method	A growth-promoting method where specific compounds are used as indicators of pathogen presence.	Oxidase test, catalase test, indole production test, methyl red, blood agar plates, motility agar, etc.	Accurate, high specificity	Slow
	Immunological based method	Uses antibodies to detect specific bacterial antigens.	Enzyme-linked immunosorbent assay (ELISA), lateral flow immunoassay, immunofluorescence assay, immunomagnetic separation, latex agglutination, immunodiffusion assays, etc.	Highly specific and can be rapid	Expensive, requires pre-enrichment steps, false positive results
	Nucleic acid based method	Detects bacteria by amplifying or identifying their DNA or RNA	Polymerase chain reaction (PCR), multiplex PCR, real-time PCR, quantitative real-time PCR (qPCR), and reverse transcriptase PCR	Highly sensitive, specific, and faster than culture methods.	Expensive, requires specialized equipment, and may not distinguish between live and dead bacteria.
Novel methods	Hybridization-based method	Uses complementary nucleic acid probes to bind to specific bacterial DNA or RNA sequences, enabling detection.	Fluorometric, colorimetric, electrochemical, and chemiluminescent	Rapid, stable, and sensitive	Requires instrumentation
	Array-based method	Involves immobilizing multiple probes on a solid surface to detect several bacterial species or genes simultaneously.	DNA microarray, alternative array-based detection	Rapid, sensitive, high accuracy, and throughput	Confusion of first-time users, non-reproducible results
	Spectroscopy technique	Uses light absorption, scattering, or emission to detect bacterial components, such as lipids, proteins, or nucleic acids.	Fourier transform infrared spectroscopy (FTIR), Raman spectroscopy, Hyperspectral imaging techniques (HSI)	Sensitive, rapid	Time-consuming, and interference with fluorescence
	Loop-mediated isothermal amplification (LAMP)	A nucleic acid amplification method that uses four sets of primers to identify six distinct zones on the targeted gene.	LAMP	Rapid, sensitive, reliable, do not require trained personnel	Low throughput
	Biosensor	Combines biological recognition elements (e.g., antibodies, enzymes, or DNA) with a transducer to detect bacteria through electrical, optical, or chemical signals.	Electrochemical biosensor (Amperometric, Voltammetric, Potentiometric, Impedimetric), Optical biosensor (Colorimetric, Fluorescent, Surface plasmon resonance (SPR) biosensor, Surface-enhanced Raman scattering (SERS)), Mass-sensitive biosensor (Piezoelectric, Magnetostrictive)	Easy, low cost, rapid, and highly selective	Still under development for commercialization in foodborne pathogen detection, and may have limitations in complex food matrices

**Table 2:** Electrospun nanofiber-based biosensors for detection of foodborne bacteria.

Target Foodborne Bacteria	Detection Method	Nanofiber Composition	Food Marix	LOD	Response Time
				(CFU/mL)	
*Salmonella Typhimurium* [51]	Chemiresistive	SU-8 photoresist	Beef	10	5 min
*Salmonella* [52]	Differential pulse voltammetry	GE-MB/Au NPs/CNFs/Chi	Full-fat milk	1.223	-
*Salmonella* [53]	Impedimetric	PU/P3ANA	-	-	-
*Salmonella*, *E. coli* [54]	Immunoassay	PCL	-	10²	12 min
*E. coli*,	Colorimetric	PLLA/anthocyanin	-	10²	-
*L. monocytogenes* [55]					
*E. coli* [56]	Colorimetric	PVA-co-PE	-	26	15 min
		Cellulose acetate butyrate		69	30 min
*E. coli* [57]	Colorimetric	PDA	-	-	30 min^−1^ h
*E. coli* [58]	Potentiometric	PAA/PVA	Water	20	-
*E. coli* [59]	Potentiometric	PAA/PVA	Orange juice	10²	<1 h
*E. coli* [60]	Conductometric	PVDC/NC	Water	61	8 min
*E. coli* [61]	Magnetic immunoassay	CN NFs/MNPs	-	67	8 min
*L. monocytogenes* [62]	Amperometric	MWCNT	Milk	1.07 × 10^2^	-
*L. monocytogenes* [63]	Impedimetric	TiO_2_	-	4.7 × 10^2^	50 min
*S. aureus*, *P. putida* [64]	Fluorescence	PAN/pVDB	-	-	-
*S. aureus* [65]	Colorimetric	MO@CNPs/Gelatin nanofibers	Cheese	-	-
*S. aureus* [66]	Colorimetric	PDA/PU	-	45 × 10^2^	-

Chi: chitosan; GE: graphite electrode, Au NPs: gold nanoparticles; CNFs: carbon nanofibers; PU: polyurethane; P3ANA: poly(m-anthranilic acid); PCL: polycaprolactone; PLLA: poly-l-lactic acid; PDA: polydiacetylene; PAA: poly acrylic acid; PVA: polyvinyl alcohol; PVA-co-PE: polyvinyl alcohol-*co*-ethylene; PVDC: polyvinylidene chloride; NC: nitrocellulose; MNPs: magnetic nanoparticles; PLLA: poly-l-lactic acid; MWCNTs: Multi-walled carbon nanotubes; TiO2: titanium dioxide; PAN: polyacrylonitrile; pVDB: poly(4-vinylphenylboronic acid-co-2-(dimethylamino)ethyl methacrylate-co-n-butyl methacrylate); MO@CNPs: moringa oil-loaded chitosan nanoparticles; PDA: polydiacetylene; PU: polyurethane.

## Data Availability

Data are contained within the article.
